# An *Anopheles stephensi* Promoter-Trap: Augmenting Genome Annotation and Functional Genomics

**DOI:** 10.1534/g3.118.200347

**Published:** 2018-08-22

**Authors:** William Reid, Kristina Pilitt, Robert Alford, Adriana Cervantes-Medina, Hao Yu, Channa Aluvihare, Rob Harrell, David A. O’Brochta

**Affiliations:** * Institute for Bioscience and Biotechnology Research, University of Maryland College Park, 9600 Gudelsky Drive, Rockville, MD 20850-3467; † Insect Transformation Facility, Institute for Bioscience and Biotechnology Research, University of Maryland College Park, 9600 Gudelsky Drive, Rockville, MD 20850-3467; ‡ Department of Plant Protection, Henan Institute of Science and Technology, East Street Huan-Lan, Xinxiang City, Henan Province 453003, CHINA; § Department of Entomology, University of Maryland College Park, 4112 Plant Sciences Building, College Park, MD 20742-4454

**Keywords:** insect genomics, malaria, transposon gene vectors, Asian mosquito

## Abstract

The piggyBac transposon was modified to generate gene trap constructs, which were then incorporated into the genome of the Asian malaria vector, *Anopheles stephensi* and remobilized through genetic crosses using a piggyBac transposase expressing line. A total of 620 remobilization events were documented, and 73 were further characterized at the DNA level to identify patterns in insertion site preferences, remobilization frequencies, and remobilization patterns. Overall, the use of the tetameric AmCyan reporter as the fusion peptide displayed a preference for insertion into the 5′-end of transcripts. Notably 183 – 44882 bp upstream of the *An. stephensi* v1.0 *ab initio* gene models, which demonstrated that the promoter regions for the genes of *An. stephensi* are further upstream of the 5′-proximal regions of the genes in the *ab inito* models than may be otherwise predicted. RNA-Seq transcript coverage supported the insertion of the splice acceptor gene trap element into 5′-UTR introns for nearly half of all insertions identified. The use of a gene trap element that prefers insertion into the 5′-end of genes supports the use of this technology for the random generation of knock-out mutants, as well as the experimental confirmation of 5′-UTR introns in *An. stephensi*.

Temporal and spatial patterns of gene expression are important phenotypes that contribute to our understanding of gene function. Molecular methods that rely on recovering and characterizing mRNA at specific times and in specific locations such as microarray- and RNAseq-based approaches are powerful methods by which gene expression patterns can be determined but the temporal and spatial resolution achievable with these approaches are limited by a number of factors. *In situ* hybridization and RNAseq technologies can achieve single-cell resolution and important improvements continue to be developed (Satija *et al.* 2015; Morris 2016). Genetic approaches referred to as “promoter-trapping” are alternative approaches for discovering and analyzing temporal and spatial patterns of gene expression (von Melchner and Ruley 1989; Rojas-Pierce and Springer 2003). These genetic approaches are attractive because users can assess the expression patterns of large numbers of genes with single cell resolution.

Promoter-traps today are transposon-based technologies in which an active transposon vector contains transgenes whose insertion into or near regions of a genome with distinct properties and functions influence the expression of the transgenes (Brickman *et al.* 2010). Current technologies used in eukaryotes are variations of gene-fusion technology developed originally in prokaryotes (Casadaban and Cohen 1979). Typically these genetic sensor technologies are designed such that when the sensor-containing transposon inserts into or near a certain functional domain a reporter gene is expressed leading to a visual output, and in some cases positive and direct selection of individuals harboring new insertions of the sensor-containing transposon are possible. The broad category of sensor systems referred to as promoter-traps include specific sensors for enhancers (enhancer-traps), promoters (promoter-traps), transmembrane proteins (secretory-traps), exons (exon-traps) and transcribed genes (polyA-traps) (Weber *et al.*, 1984; von Melchner and Ruley 1989; Duyk *et al.*, 1990; Krizman and Berget 1993; Mitchell *et al.*, 2001).

Transposons designed to function as an enhancer-trap typically contain a reporter gene (*e.g.*, a fluorescent protein or the Gal4 transcription factor) regulated by a basal promoter which in the absence of an enhancer, is insufficient to result in detectable levels of the reporter gene product. Integration of the element within the domain of an active enhancer will result in the *cis*-activation of the basal promoter and expression of the reporter gene. While of great utility, enhancer-trapping does not enable the easy identification or isolation of the regulatory sequences responsible for the expression of the reporter gene since enhancers can act at considerable distances from the genes they are regulating. Alternatively, for insertion mutagenesis and gene identification purposes, promoter-, secretory-, exon- or polyA-traps are required (Friedel and Soriano 2010).

Promoter-traps consist of a transposon with a reporter gene lacking promoter and enhancer sequences. Instead, a 3′ splice acceptor site is located at the 5′ end of a reporter’s open reading frame. Integration of a promoter-trap element into an intron of an actively transcribed gene can result in splicing of the reporter-containing exon and a fusion protein, provided the phases of the exon 5′ of the targeted intron and the reporter gene are the same. In addition to reporting on the activity of the promoter of the targeted gene, insertions and subsequent alternative splicing often result in hypomorph or null alleles of the targeted gene. Consequently, forward genetic screens using transposons configured as promoter-traps can be used to identify genes, reveal their temporal and spatial patterns of expression and at the same time create null or hypomorph alleles that can help in determining the function of the targeted gene.

Extensive use of promoter-trapping technologies in popular insect systems such as *Drosophila melanogaster*, Bombyx mori and Tribolium castaneum has led to the development and maintenance of large collections of lines of insects with transposon insertion mutations (Bellen *et al.*, 1989; Trauner *et al.*, 2009; Tsubota *et al.*, 2014).

While these lines are valuable community resources, maintaining large collections of mutant lines of other insects tends to be less feasible for most other insect systems. Nonetheless, promoter-trapping technologies are still very useful in other insect systems. Promoter-trap screens could be performed and the resulting mutants characterized by photo-documenting temporal and spatial patterns of reporter gene expression (visible phenotypes) as well as determining the location of the integrated promoter-trap element. After this initial characterization is completed the line could be discarded and any subsequent interest in the gene or the original promoter-trap allele could be satisfied by recreating the mutation using RNA-guided endonuclease-based gene integration technologies.

Here we report on a *piggyBac*-based promoter-trap designed and constructed for use in mosquito genomes and report on results from a forward genetic screen conducted in *An. stephensi* involving approximately 125,000 mosquitoes. A total of 620 promoter-trap events were observed, of which 73 were characterized for insertion into a gene. These events enabled existing gene models to be confirmed in some cases and in others for new models to be developed. Notably the annotation for the 5′-UTR regions for genes, and the expected promoter regions of genes, which in roughly half of the promoter trap insertions, was distal to the +1 of the transcript ORF and terminated within the 5′-UTR. The results from our study enhance the current annotation of the *An. stephensi* genome, and also provide insight for the design of putative ubiquitous and tissue-specific promoters within mosquitoes.

## Materials and Methods

### DNA

**Figure 1:**
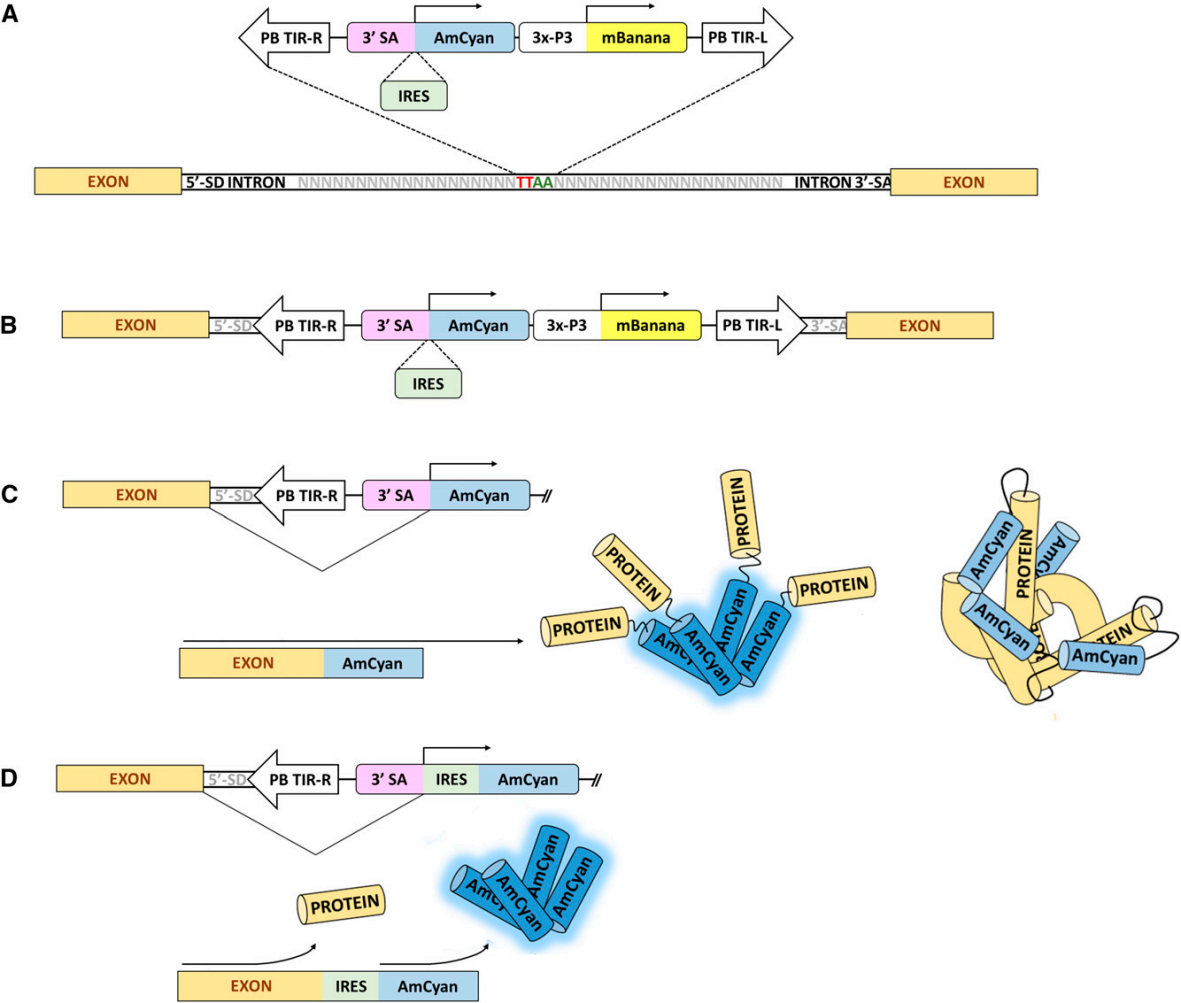
Schematic of how the promoter trap element functions. A) Insertion of the piggyBac promoter trap element into the TTAA piggyBac insertion site contained within an intron. B) The transcript strandedness must be consistent with that of the AmCyan ORF. C) Splicing of the promoter trap element and subsequent tetramer formation of the AmCyan protein to produce a functional AmCyan reporting fluorescent protein. Longer protein tags from the endogenous *An. stephensi* transcript may potentially interfere with the quaternary functional structure of AmCyan. D) Splicing of the promoter trap element containing the IRES element allowing for expression of the AmCyan protein independently of the endogenous peptide tag may alleviate potential interference of a peptide tag on the quaternary structure formation of AmCyan.

*PB3′SA-0AmCyan* and *PB3′SA-IRESAmCyan*: These are promoter-trap vectors constructed from *piggyBac* elements consisting of 671bp and 690bp of *piggyBac’s* 5′ and 3′ terminal sequences, respectively (GenBank: J04364.2). Using the Gateway cloning system (Invitrogen, Carlsbad, CA) a dominant visible marker and a promoter-trap reporter were inserted between the terminal sequences of *piggyBac*. The dominant visible marker consisted of *mBanana* (Clonetech, Mountain View, CA) with or without a nuclear localization signal under the regulatory control of the neural-specific promoter *3XP3* (Horn and Wimmer 2000) with a 3′ UTR from the SV40 VP1 gene (Reddy *et al.* 1978). The promoter-trap reporters in these vectors consisted of the open reading frame of the fluorescent protein gene *AmCyan* (Clonetech) with a 3′ UTR from the SV40 VP1 gene. In *PB3′SA-0AmCyan* the *AmCyan* open reading frame had a 3′ splice acceptor site resulting in a phase-0 splice of *AmCyan* and the targeted transcript. *PB3′SA-IRESAmCyan* was identical to *PB3′SA-0AmCyan* except that after the 3′ splice acceptor site there was the internal ribosomal entry site (*IRES*) from Drosophila C dicistrovirus (Carter *et al.* 2008) followed by the open reading of *AmCyan* and a SV40 3′ UTR. The promoter-trap reporters were inserted into the *piggyBac* vectors such that the 3′ splice acceptor sites were adjacent to the 3′ terminal sequences of *piggyBac* (Figure 1). The promoter trap element will display only if it lands within the transcribed region of the genome in the proper orientation. The IRES element was added to the promoter trap construct to address two concerns: 1) that the splice event may occur in a phase I or II intron, leading to a frameshift of AmCyan, and 2) to determine insertion preference of the element since AmCyan functions as a tetramer, and may report easier if it has little-to-no associated peptide tag from the transcript (Figure 1). The latter condition of having little-to-no associated peptide tag is ideal for a promoter trap since insertion into a 5′-UTR intron would allow for the downstream sequence of the intron/UTR to be excluded from the predicted promoter since functionality could be achieved using only the intron up to the 3′-SA. Since the IRES allows for expression independent of the peptide within the transcript, it can insert into any intron and allow for the expression of AmCyan without a protein tag, allowing for easier quaternary structure formation, while the 3′-SA absent the IRES would require that any tagged peptide not interfere with AmCyan tetramerization. Thus, we anticipated that the 3′SA absent IRES would have a preference for 5′-end insertion within the transcript, while the IRES + 3′SA would function independently of this requirement.

Each of these vectors was attached to a plasmid backbone containing the ColE1 origin of replication along with genes conferring antibiotic resistance (ampicillin, kanamycin or zeocin).

#### PB1.5vasaPBtnpsase:

**Figure 2:**
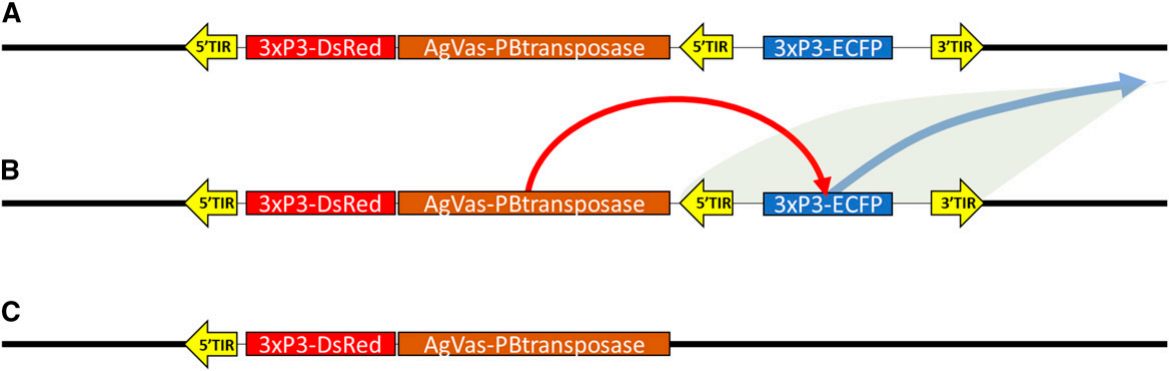
Schematic of how the self-marooning piggyBac transposase element functions. A) The cassette for the piggyBac transposase self-integrates into the genome. The marooning construct is flanked by 5′ and 3′ piggyBac end and contains an internal 5′ end flanking a second fluorescent marker. B) Expression of the transposase results in the remobilization of the ECFP fluorescent marker. C) The vasa-driven piggyBac transposase construct is marooned without a 3′ piggyBac end and is identifiable by the presence of the DsRed fluorescent marker and the absence of the ECFP fluorescent marker.

This is a *piggyBac* vector used to introduce the *piggyBac* transposase transcription unit under the regulatory control of the promoter from the *Anopheles gambiae vasa* gene and was designed based on the integrated vector stabilization strategy described by Handler *et al.* (2004) (Handler *et al.* 2004)(Figure 2). This vector contains a small non-autonomous *piggyBac* element (*pXL-BacII-3xP3-ECFP*) with the fluorescent protein gene *Enhanced Cyan Fluorescent Protein* (ECFP) under the regulatory control of the promoter *3XP3* (Li *et al.* 2005). In addition, flanking the 3′ *piggyBac* inverted terminal repeat of pXL-BacII-3xP3-ECFP is a copy of the dominant visible marker *DsRed* (Clonetech, Mountain View, CA) under the regulatory control of the promoter *3XP3* (Horn and Wimmer 2000) with a 3′ UTR from SV40 (Reddy *et al.* 1978), as well as a 2.3 kb fragment containing the promoter of the *An. gambiae vasa* gene attached to the 5′ end of a *piggyBac* element. The *vasa* promoter was amplified and cloned from the Nguso line of *An. gambiae* as described by Papathanos *et al.* (2009) (Papathanos *et al.* 2009). This results in an element having two copies of the 3′ terminal sequences of *piggyBac*, one copy of the 5′ terminal sequences of *piggyBac* as well as a *piggyBac* transposase gene expressed in the germline. This vector is attached to a plasmid backbone containing the ColE1 origin of replication along with an ampicillin resistance gene.

### Mosquitoes

Adult mosquitoes were maintained at 29° and 80% relative humidity with continuous access to an aqueous solution of 10% sucrose. Larvae were reared in deionized water at 29° and provided with pulverized fish food (TetraMin Tropical Flakes) *ad libitum* and the density of larvae was adjusted to minimize larval development times. Mated adult females were presented with adult mice from which they obtained a blood-meal so they could successfully complete oogenesis. The use of mice was with the approval and oversight of the University of Maryland, College Park’s Institutional Animal Care and Use Committee (IACUC) operating under the National Institutes of Health’s Office of Laboratory Animal Welfare guidelines.

All of the transgenic insects used in this study were created using the services of The University of Maryland College Park Institute for Bioscience and Biotechnology Research’s Insect Transformation Facility.

#### SDA 500:

This is a wild-type strain of *An. stephensi* selected for susceptibility to infection by *Plasmodium falciparum* (Feldmann and Ponnudurai 1989).

*UMITF-PB-M5^DsRed^* (referred to as hspPB): This is a previously described transgenic line of SDA 500 with the *piggyBac transposase* transcription unit under the regulatory control of the promoter from *D. melanogaster’s hsp70* gene and the red fluorescent protein gene *DsRed* under the regulatory control of the synthetic promoter *3XP3* (O’Brochta *et al.* 2011). Expression of *piggyBac transposase* in this line can remobilize *piggyBac* elements in the soma and germ-line (O’Brochta *et al.* 2011).

*UMITF-vasaPB ^DsRed^* (referred to as vasPB): This is a transgenic line of SDA 500 created using the vector *PB1.5vasaPBtnpsase*. Primary transgenic insects containing the vector *PB1.5vasaPBtnpsase* were selected that simultaneously expressed *ECFP* and *DsRed* and were subsequently mated to SDA 500 of the opposite sex. Progeny from this cross that expressed only *DsRed* because of the excision of *pXL-BacII-3xP3-ECFP* from *PB1.5vasaPBtnpsase* were selected and used to create the line *UMITF-vasaPB ^DsRed^*. Excision of *pXL-BacII-3xP3-ECFP* from *PB1.5vasaPBtnpsase* results in the loss of the only functional *piggyBac* 5′ inverted terminal repeat in this vector, leaving the *vasa*::*piggyBac transposase* transgene and the associated *3XP3*::*DsRed* genetic marker stably located in the original site of integration. This line expresses *piggyBac transposase* in the germ-line.

*DO-05-13M1*, *DO-05-13F1C*, *DO-05-13F1D*, *DO-05-13F2A*, *DO-05-13F2B* (referred to as M1, F1C, F1D, F2A, F2B respectively): These were transgenic lines of SDA 500 with *PB3′SA-0AmCyan* (line F1C) or *PB3′SA-IRESAmCyan* (lines M1, F1D, F2A, F2B) inserted at single loci and maintained as homozygotes. None of these lines displayed any detectable *AmCyan* expression in the larval or adult stages, however the 3xP3::mBanana marker was visible in all lines.

### Genetic Crosses and Screens

To initiate a promoter-trap screen, the *piggyBac* promoter-trap element was remobilized in the *An. stephensi* genome by crossing virgin females containing a promoter-trap element (M1, F1C, F1D, F2A, F2B) to males from the transposase expressing lines hspPB or vasaPB *en masse* (2:1 female:male sex ratio). Females heterozygous for the promoter-trap element (expressing *mBanana* or nls-*mBanana*) and the transposase-expressing transgene (expressing *DsRed*) were mated *en masse* to SDA500 males (2:1 female:male sex ratio). Larvae (3^rd^ or 4^th^ instar) and/or adults with the promoter-trap element (*mBanana* or nls-*mBanana*) were screened for the presence of non-parental patterns of *AmCyan* expression indicating that the promoter-trap element was now in a new location and was being transcribed and translated. The frequency of remobilization was estimated by dividing the number of *AmCyan*-expressing individuals over the total number of mosquitoes screened. The *AmCyan* expression patterns in individuals with a promoter-trap event were documented photographically. These individuals were either saved and lines established by outcrossing them to SDA500 and screening for and retaining progeny expressing the marker genes *mBanana* or nls-*mBanana*, or they were frozen on dry ice and stored at -80° for RNA/DNA extraction and subsequent analysis.

### Integration site identification

To identify the integration site of the promoter-trap element in line M1, genomic DNA was extracted following Ashburner (1989) and used in a Splinkerette PCR reaction as described below (Ashburner 1989; Potter and Luo 2010).

For the remaining promoter-trap lines F1C, F1D, F2A, F2B, total RNA and DNA were extracted from individual mosquitoes using TRIZol (Life Technologies, Carlsbad CA) according to the manufacturer’s instructions. RNA was isolated first, followed by back-extraction of DNA mediated by the addition of TNES-6U (Triant and Whitehead 2009).

To perform Splinkerette PCR, up to 1 µg of DNA was digested with either *Bst*YI or *Bgl*II and the resulting fragments were ligated to Splinkerette adapters (Potter and Luo 2010). Primers specific to the 5′ and 3′ terminal inverted repeats of the *piggyBac* transposon were amplified through a series of semi-nested PCRs in which the PCR products contained the end of *piggyBac* and genomic DNA immediately flanking the element. Splinkerette PCR products were gel-purified using the Qiagen gel extraction kit (Qiagen, Germantown MD) and their sequences determined using the method of Sanger *et al.* (Sanger *et al.* 1977) using either the 5′PB-seq primer or the 3′PB-seq primer. The primers used for Splikerette PCRs are listed in Table S1. DNA sequencing services were provided by Macrogen USA (Rockville MD). Following DNA sequencing, the presence of *piggyBac* was confirmed and the genomic insertion site was mapped to the *An. stephensi* genome (strain SDA500 version: AsteS1) (Jiang *et al.* 2014) using nucleotide BLAST (Altschul *et al.* 1990).

The RNA from mosquitoes with promoter-trap events was pooled to form a master-pool of RNA consisting of up to 1 µg RNA from each individual (75 from DO-05-13-F2A; 35 from DO-05-13 F1D; 73 from DO-05-13 F1C). The RNA was re-precipitated with one volume of isopropanol and 1/10 volume of 3M NaOAc (pH 5.2). The resulting RNA pellet was washed twice with 80% ethanol and re-suspended in nuclease-free water. Thirty micrograms of the pooled RNA was treated with 30U of DNAse I (Promega, Madison WI), re-extracted using TRIzol, precipitated with one volume of isopropanol and the precipitate was washed twice with 80% ethanol. The precipitated RNA was dried and re-suspended into nuclease free water to a final concentration of 1 µg/µL. This RNA was sequenced using Illumina RNASeq with polyA selection (paired-end 50 nt reads) using the Illumina TruSeq mRNA library kit (Illumina, San Diego CA) by the Hudson Alpha Institute for Biotechnology (HAIB) (Huntsville, AL).

Preliminary analysis of the RNASeq data including initial base-calling and conversion of the read data to fastq format was conducted by the HAIB. The raw reads obtained from the HAIB were trimmed of adapter and low quality sequences using Trimmomatic (v0.35), and mapped to the *An. stephensi* genome (AsteS1) using Tophat2 (Kim *et al.* 2013; Bolger *et al.* 2014) . Splice junctions and introns were predicted using Cufflinks2 and visually confirmed for each genomic Splinkerette insertion using the Integrative Genomics Viewer to confirm that the genomic insertion orientation of the promoter-trap element was correct with respect to the orientation of the predicted transcript (Robinson *et al.* 2011; Trapnell *et al.* 2012; Thorvaldsdottir *et al.* 2013) . Insertion sites that were present on scaffolds linked to chromosomes were then mapped to chromosomes based on the physical map for *An. stephensi* (Sharakhova *et al.* 2010) . Individual paired end reads were also inspected for fusion events of *An. stephensi* transcripts and the IRES element or the AmCyan open reading frame, using grep exact pattern searching.

### Data Availability Statement

Short read data from this manuscript have been submitted to the SRA database at NCBI as accession number SRR3195175 and the NCBI GEO database as accession number GSM2076255, which contains the gtf file that predicts the introns not present in the ab initio AsteS1 version of the *An. stephensi* genome. The sequences from the genomic insertion sites have been deposited to the GSS database at NCBI as accessions KS331897 - KS331997. Supplemental material available at Figshare: https://doi.org/10.25387/g3.6995042.

## Results

### Promoter-trap element remobilization, screening, and frequency of promoter-trap remobilization events

In total five lines of *An. stephensi* containing the piggyBac promoter-trap element were established through microinjection (referred to as M1, F1C, F1D, F2A, F2B), representing five distinct starting loci for piggyBac remobilization (Table 1). Since these lines displayed no detectable *AmCyan* expression in the larval or adult stages, a positive promoter-trap event was considered to have occurred if the AmCyan fluorescent reporter was visible following remobilization of the piggyBac element. That is: the promoter-trap element had been relocated from an initial non-reporting insertion site into a transcript, resulting in the expression of AmCyan. Individual promoter-trap remobilization events were photodocumented and the DNA and RNA were extracted to identify the promoter-trap element insertion site, where possible. In total, >124,00 mosquitoes were screened for promoter-trap events for the five starting lines M1, F1C, F1D, F2A, and F2B, resulting in a total of 620 observable promoter-trap remobilization events (Table 1). Overall, all starting lines resulted in positive promoter-trap remobilization events, however the frequencies varied greatly. Two experimental parameters with the potential to contribute to variation in the rate at which promoter-trap events are recovered include the genomic starting position of the promoter-trap element (lines) and the source of *piggyBac* transposase (so called “jumpstarter” elements). There is some evidence that both parameters were important in this study. For example, the transposition activity of the promoter-trap element in line F2B showed reduced transposition activity, probably resulting from the influence of genomic location on *piggyBac* remobilization activity, an effect well documented in *Drosophila melanogaster* (Table 1) (Esnault *et al.* 2011). The promoter-trap element in line F2B is located in cytogenetic position 6A on the X chromosome and resulted in the lowest frequency of promoter-trapping events regardless of the jumpstarter element used to remobilize the element (hspPB or vasPB). Promoter-trap events originating from transposition of the promoter-trap element in F2B were recovered at approximately one tenth of the overall frequency of recovery of promoter-trap events in this experiment, limiting the utility of this line for conducting genetic screens. The element in line F2A was the source of the greatest number of promoter-trap events although its insertion site location could not be precisely located on a chromosome because of the limitations of the current assembly of the *An. stephensi* genome. Overall, one needed to screen approximately 200 individuals before a promoter-trap event was detected.

**Table 1:** Remobilization frequency of the promoter trap element within the genome of *An. stephensi*

Line	Chromosome locus	Number of individuals screened	Number of promoter traps	Remobilization Frequency (%)
DO-05-13-F1C	3R (34A)	37500	121	0.32
*DO-05-13-F1D	3L (45C)	22613	68	0.30
*DO-05-13-F2A	n/a	34918	274	0.78
*DO-05-13-F2B	X (6A)	11847	7	0.06
*DO-05-13-M1	3L (46C)	17500	150	0.86
	TOTAL:	124378	620	

* Promoter trap lines containing the upstream IRES element.

### Phenotypes

Upon insertion into a transcript, the AmCyan fluorescent reporter was visible in both tissue-specific, and multiple tissue patterns, indicating that the promoter-trap element had landed in a transcript with a similar tissue-type expression pattern. A total of 232 phenotypes were recorded for larvae, while 71 were observed for adults.

### Larvae

**Figure 3:**
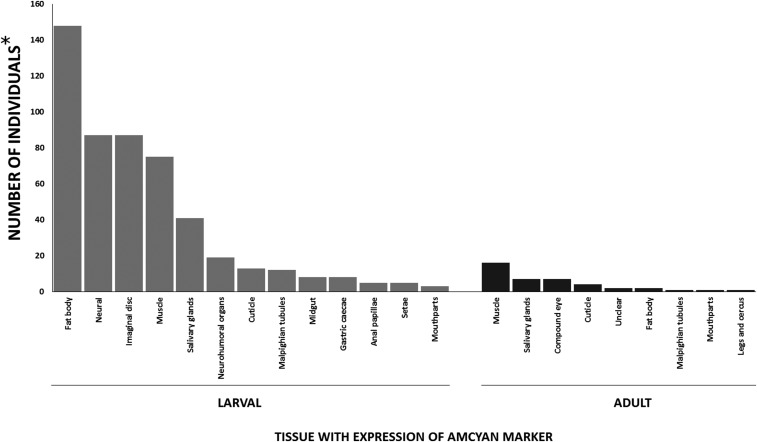
Distribution of observed promoter trap phenotypes in *An. stephensi* adults and larvae. *The number of individuals records the observed phenotype for all individuals displaying AmCyan expression mwhere individuals observed with multiple tissue phenotypes are recorded for each tissue type present in the individual.

**Figure 4:**
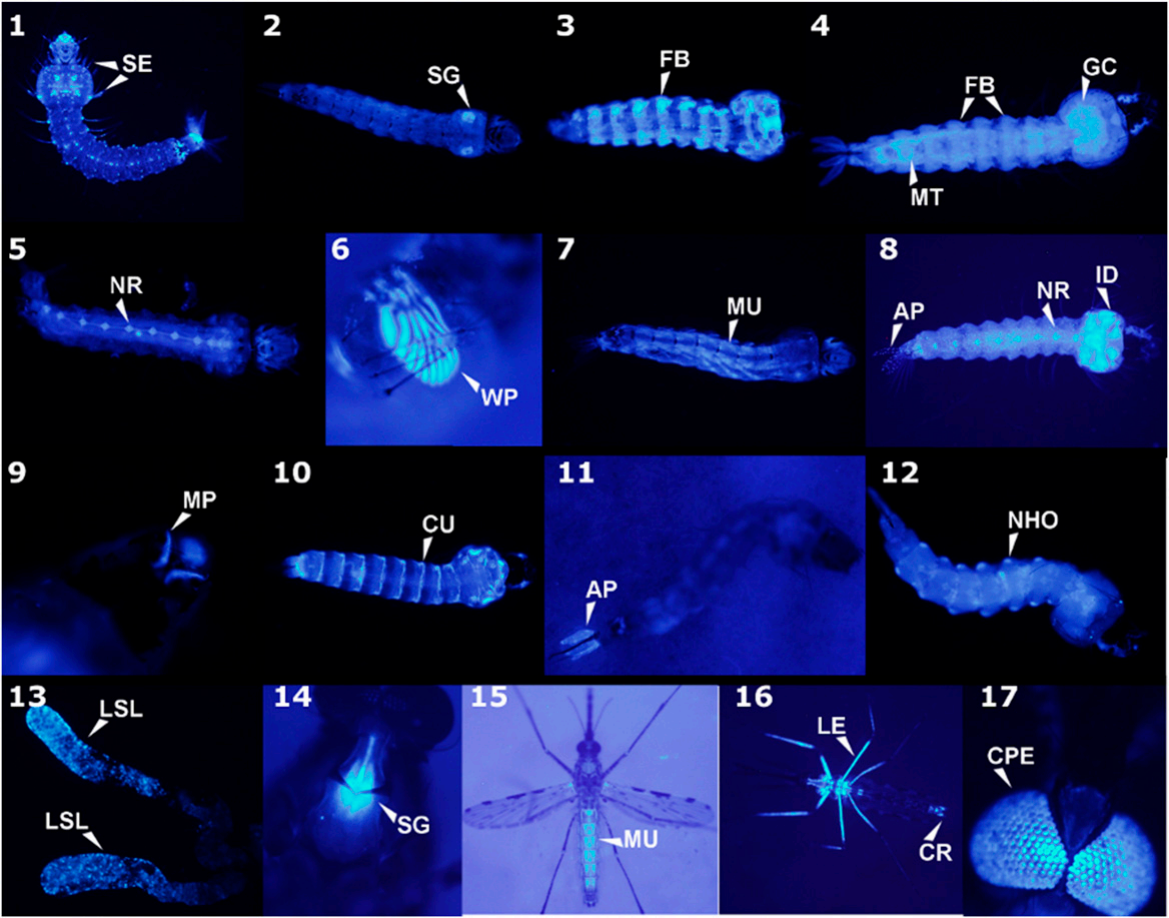
Tissue-specific promoter trap AmCyan expression in *An. stephensi* larvae (panels 1 – 12) and adults (13 – 17). AP = anal papillae, CPE= compound eye, CR= cercus, CU= cuticle, FB= fat body, GC= gastric caecae, ID= imaginal disc, LE= legs, LSL= lateral salivary lobes, MP= mouthparts, MU= muscle, NHO= neurohumeral organs, NR= neural, SE= setae, SG= salivary glands, WP= wing pad.

The promoter trap positive events predominantly resulted in multiple tissue type expression with only 19% of the 232 total events recorded displaying single tissue-specific patterns (Figures 3, 4). Among the larval promoter trap events, 29% occurred in the fat body (148 events, 21 of which were fat body specific) and represented the most frequently observed tissue-type expression pattern. Other major patterns identified in the larvae were neural, imaginal disc, muscle, and salivary glands, which represented 17, 17, 14, and 8% of all larval promoter traps, respectively. Minor tissue display promoter trap events occurred in the neurohumeral organs, cuticle, Malpighian tubules, midgut, anal papillae, gastric caecae, setae, and mouthparts.

### Adults

A total of 30 of the 71 phenotypes observed among the adult promoter trap events reported fell within three of the commonly observed tissues in the larval promoter traps: muscle, salivary gland, compound eye (neural) (Figures 3, 4). Notably, a greater reduction in tissue-specific fat body gene trap events was observed. This could be due to a reduction in gene expression in the adult fat body, where the larval *An. stephensi* have a highly metabolically-active fat body in order to produce high levels of storage lipids and proteins, whereas basal gene expression in the adult fat body may be lower. Martínez-Barnetche *et al.* (2012) found that only 35 of 16,669 transcripts were specifically expressed in the fat body (Martínez-Barnetche *et al.* 2012). Also, in part, the lower observation of fat body promoter traps in adults could be due to the darker cuticle of the adults compared to the larvae, where AmCyan expression in the adults would need to be higher in order for visual confirmation of the promoter trap event. In addition, due to the simplicity of larval screening, more individuals were screened as larvae, than for as adults.

### Locating Promoter-Traps

Locating promoter-trap elements is essential in order to connect genotypes and the reporter gene-expression phenotypes associated with those genotypes. Two methods were used in this study, one at the DNA level and one at the RNA level, with varying degrees of success. First, of the 620 gene trap events detected in this study, 232 were used for integration site analysis, and of these, 73 insertions were successfully characterized using the PCR-based method to selectively amplify a small terminal region of the *piggyBac* transposon used to vector the promoter-trap reporter cassette and the genomic DNA immediately flanking the ends of the element (Table S2). We found that only about 25% of the 232 integration events recovered could be localized successfully using only this method. Of the insertions, nearly half were located within 5′-UTR regions not annotated in the *ab initio* genome models.

The second method used to identify the location of the integrated promoter-trap element relied on the sequence analysis of the transcriptome. In this case, short-read Illumina RNA sequence data were examined for the presence of sequences corresponding to the reporter gene found on the promoter trap element. This method was also somewhat inefficient because from the transcriptomes of some 200 larvae, each representing a single promoter-trap event, we were only able to definitively locate the targeted integration site of 17 events. While the low recovery was likely due to relatively low transcript expression following sample pooling, we were able to recover and document several events at the RNA level that confirmed the functionality of the promoter trap elements at the transcript level. Following mapping of the reads to the *An. stephensi* genome, the unmapped reads (17,167,314 singletons) were then extracted from the trimmed reads and remapped to the *An. stephensi* genome with the inclusion of a ‘pseudoscaffold’ consisting of the entire *piggyBac*-end flanked IRES-containing promoter trap element. A total of 13,967 reads mapped to the promoter trap element (Figure S1). The majority of the reads mapped to the mBanana and AmCyan fluorescent reporters (9,695 and 3,637 reads respectively), with additional coverage of the 3xP3 promoter region (586 reads) likely due to read-through from the AmCyan transcript. In addition, some coverage was present among the *piggyBac* ends (18 and 25 reads for the left and right ends, respectively) supporting the observation of integration of the promoter trap element into exonic sequence. The exonic insertions possibly resulted in readthrough of the promoter trap element from the right *piggyBac* end to the SV40 terminator sequence. Finally, the read coverage for the IRES sequence was low and contained only five reads. Since the 3′-splice acceptor for the IRES-containing promoter trap element is ∼200 bp upstream of the open reading frame of AmCyan, and since this construct was positive for AmCyan reporting promoter traps at a frequency greater than the 3′-splice acceptor alone, we postulate that there were additional unexpected splice acceptor sites upstream of the AmCyan ORF. To identify putative transcript and promoter-trap fusion events, the 5′-end of the AmCyan sequence was directly pattern searched against the *An. stephensi* genome and no exact match within the *An. stephensi* genome was found for the initial 14 nucleotides of the AmCyan open reading frame (in both orientations). Since no match was present in the genome, we reasoned that Illumina tags containing an exact match to the AmCyan open reading frame (minimum of 14 nucleotides) that were connected to sequence from *An. stephensi* represented true promoter trapped events at the RNA level. We further put two limits on these tags. First, the orientation of the AmCyan tag needed to run toward its mate pair, and the mate pair had to be an exact match (minimum of 47 nucleotides) to the AmCyan nucleotide sequence, and second, the *An. stephensi* tag needed to be at least 15 nucleotides long. After setting these limits, we identified a total of 145 paired end reads (Table 2; Table S3). Of these reads, 112 contained exact matches to the remapped RNASeq predicted splice junction positions, showing a splicing event corresponding to the 3′-splice acceptor without the IRES-containing element (NCBI GEO supplemental file GSE78771_RAW.tar). The 33 reads that did not have exact matches to the *de novo* splice donor sites shared a common 15 nucleotides common to the IRES-containing promoter trap element and were immediately upstream of the +1 start site for the open reading frame of AmCyan. Of these 33 reads, 14 of them had a tag of at least 14 nucleotides (beyond the 15 IRES and 14 AmCyan ORF nucleotides) and could be blasted to the *An. stephensi* genome to assess if they fit the model for the promoter trap element. A total of eight of the reads had perfect matches to the *An. stephensi* genome (with four unique occurrences) and formed a junction that fused with the 15 nucleotides upstream of the +1 of AmCyan, suggesting that a sequence present within the +50 amino acids of the IRES sequence contained a splice junction since the splicing of the *An. stephensi* sequence occurred at a canonical NGA site within the promoter trap element.

**Table 2:** Occurrences of resolvable splicing events of the promoter trap element using RNASeq analysis and their respective insertion locations in the *An. stephensi* genome

Construct	Transcript tag*	# occurrences	Scaffold insertion	Junction location
Non-IRES	ASTE001888	1	KB664416:466299	5′-UTR
	ASTE003097	3	KB664418:103665	Intron 1 of 4
	ASTE005963	1	KB664733:36186	5′-UTR
	ASTE006277	93	KB664587:103868	5′-UTR
	ASTE006314	1	KB664288:636233	5′-UTR
	ASTE006629	2	KB665199:435749	5′-UTR
	ASTE008287	1	KB664477:412329	5′-UTR
	ASTE011209	1	KB664576:155320	Intron 5 of 5
IRES	ASTE000126	1	KB664954:1365602	Intron 2 of 2
	ASTE000270	1	KB664732:782272	5′-UTR
	ASTE000780	4	KB665232:679279	5′-UTR
	ASTE003737	1	KB664450:281209	Intron 2 of 2
	ASTE006277	30	KB664587:103868	5′-UTR
	ASTE008914	1	KB664428:218959	5′-UTR
	ASTE009536	3	KB664400:665982	5′-UTR
	ASTE010450	1	KB664844:68028	Intron 1 of 10
	ASTE015161	2	KB664456:135953	5′-UTR

* Tags were a minimum of 14 nucleotides in length and confirmed manually to the RNASeq mapped data to confirm the junction. The transcript accession number represent the *An. stephensi* genome strain SDA500, version 1, www.vectorbase.org.

### Patterns of Transposition

**Figure 5:**
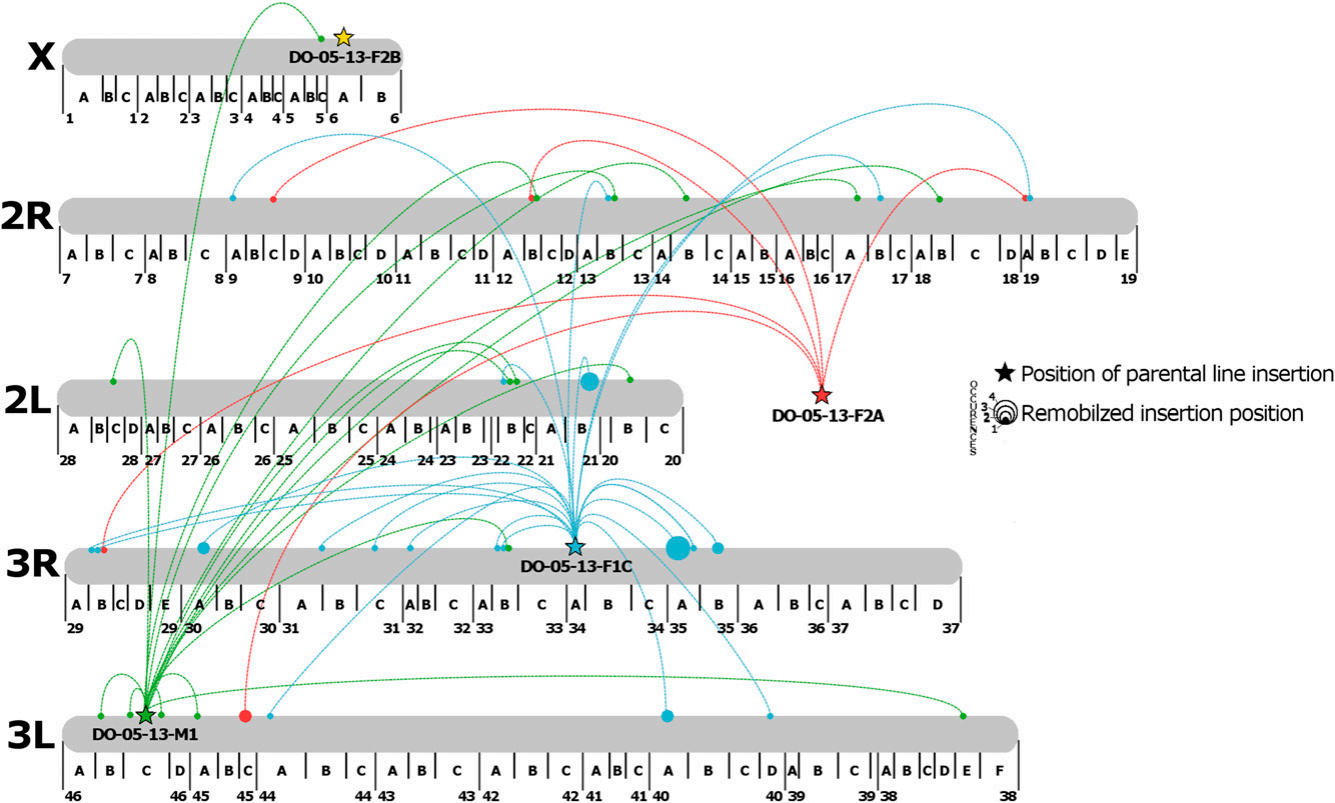
Visualization of the physical remobilization of the promoter trap elements throughout the *An. stephensi* genome. Larger dots indicate multiple hits to the same locus. Promoter trap line DO-05-13-F2A could be mapped to the *An. stephensi* genome, however the mapped scaffold did not have a corresponding chromosomal locus. In addition, remobilized promoter trap events that were identified within the *An. stephensi* genome, but could not be assigned to a chromosome are not shown.

Of the 73 promoter-trap events mapped to the *An. stephensi* genome, 49 of them were present in scaffolds that had assignment to the physical map for the mosquito. These 49 promoter-trap events were mapped to positions on the four chromosomes of *An. stephensi* (Figure 5). Of the 28 events arising from the transposition of the element in line F1C located at position 34A on chromosome 3, a disproportionate number reinserted on chromosome 3R, showing that the non-IRES element exhibited local hopping (χ^2^ = 26.28,4; *P* < 0.001). Conversely, the IRES containing elements, M1 exhibited random hopping throughout the genome (χ^2^ = 5.25,4; *P* > 0.1)., which could have been due to the initial insertion sites of the elements, or possibly due to the larger length of the IRES containing promoter trap The F2A line could not be tested for random hopping because the insertion location could not be assigned to chromosome, while the F2B line could not be tested due to the few remobilization events recorded. In addition, the F2A and M1 lines did not show a preference for chromosome 3R, suggesting that the local hopping observed for F1C was due to events other than an overall preference for *piggyBac* insertion onto chromosome 3R.

### Insertion preference within transcript

**Figure 6:**
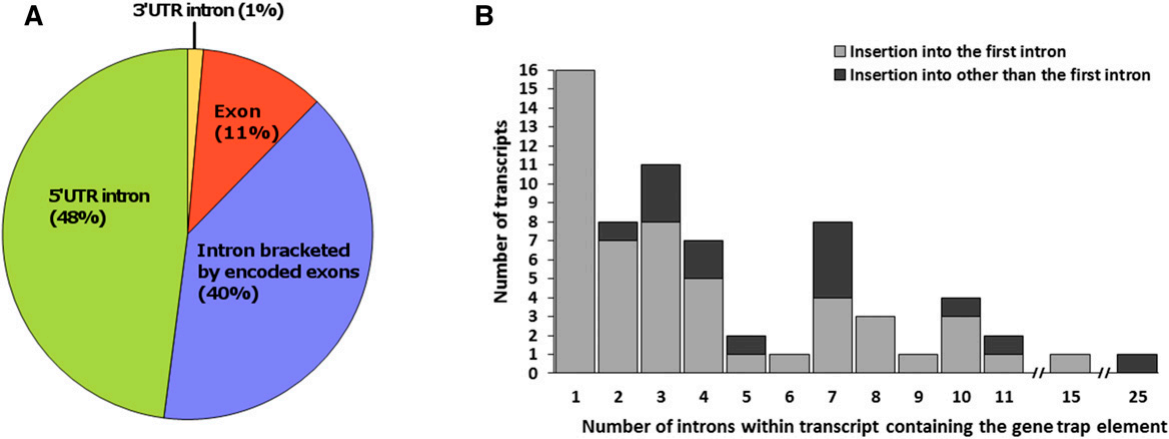
Insertion preference of the promoter trap element for the DO-05-13-M1 line within transcripts of the *An. stephensi* genome. A) location within transcript. B) Intron location of the promoter trap element in the context of the total number of introns present in the transcripts for which the promoter trap elements were positive.

**Figure 7:**
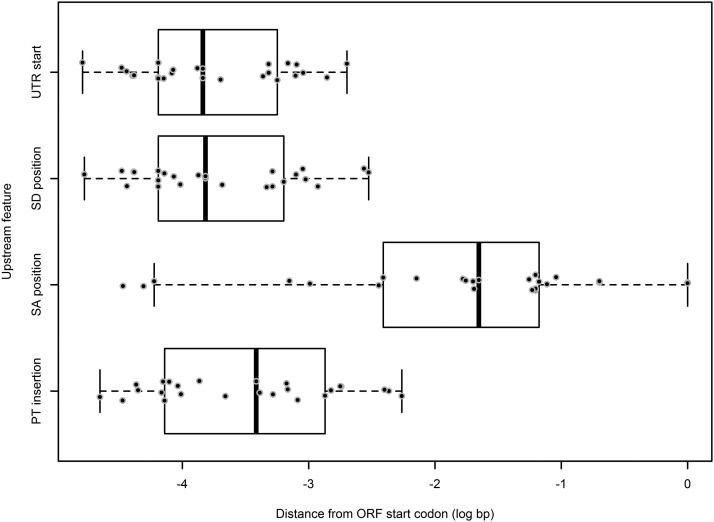
Scatter and box plot for the locations of the 5′ upstream elements of transcripts into which the promoter trap inserted into 5′-UTR introns in An. stephensi. UTR = untranscribed region, SD = splice donor, SA = splice acceptor, PT = promoter trap. The outer whiskers of each box represent the first and fourth quartiles, the box represents the data from the second to third quartiles, with the black bar within the box representing the median value for the data.

Overall, a significant majority of observable (fluorescence reporting) insertion events (51 of 65 introns; χ^2^ = 122.76,24; *P* < 0.001) occurred within the first intron within a transcript- either a 5′-UTR intron, or the first intron within the coding region compared to all other introns within the genes combined (Figure 6). In addition, 48% of the 73 mapped insertions occurred within a 5′-UTR intron, where the median upstream insertion distance for the promoter trap construct relative to the +1 start of the transcript open reading frame (ORF) was 2.6 kb (Figure 7). In addition, the distances for the upstream insertion distance of the promoter trap from the +1 start of the ORF ranged from 183 to 44882 bp (Figure 7), which indicated that the promoter regions for the identified genes within *An. stephensi* varied greatly in length. Eight of the insertions displayed a non-canonical behavior and were inserted into exons, while one insertion was identified within the 3′-UTR of a transcript.

## Discussion

The goal of our work was to develop a remobilizable genetic element that fluoresced when incorporated into the promoter proximal region of a transcript. In addition, this could allow for the identification of tissue or temporal specific putative promoter regions. A main question in the determination of a promoter sequence is the length of the upstream region required in order to allow for gene expression. While the promoter sequence may also require distal enhancers, an understanding of the actively transcribed region is helpful. To this end, the promoter trap elements designed in our study are ideal for capturing the 5′-end of transcripts. Promoter selection is often performed by selecting the upstream 1000 – 2000 bp of the +1 start codon of the gene. In our study, we identified that in *An. stephensi*, the promoter regions for many genes may be further than 2000 bp upstream from the +1 of the ORF of genes, and that the actual transcription start site was in some cases >44000 bp upstream of the +1 of the ORF. In addition, we identified that the entirety of some 5′-UTR introns be do not need to be retained for functional gene expression, which could allow for the selection of shorter promoters when using a native promoter to drive a transgene in *An. stephensi*. The use of promoter trapping is beneficial in the annotation of not only promoters, but also the transcriptional start sites of genes. For example, half of the genes identified by the Splinkerette method, and 12 of the 14 AmCyan fusion transcripts identified by RNA-Seq were fusions of AmCyan to the leading 5′-UTR exon of transcripts within the *An. stephensi* genome, demonstrating that the sequence downstream of the 3′-SA location within the 5′-UTR intron was not required for gene expression, and placing the reporting ORF closer to the transcriptional start site. This information can expedite the discovery of promoters within the *An. stephensi* genome, because it helps define a minimal downstream placement of the +1 of the ORF within 5′-UTR introns, however we recognize that since our promoter trap element was biased toward entry into 5′-UTR introns, it may be less suitable to trap promoters of genes requiring that the promoter region extend completely to the +1 of the ORF of the gene. Some genetic elements are known to have insertional biases. For example, murine leukemia virus displays a strong bias toward insertion proximal to the promoter regions, while human immunodeficiency virus inserts throughout transcripts (De Palma *et al.* 2005) . The *piggyBac* transposon has previously been found to have an insertion bias into the 5′-end of a transcripts (Häcker *et al.* 2003; Bonin and Mann 2004; Wilson *et al.* 2007; Wang *et al.* 2008; Liang *et al.* 2009). We found similar results in our study, where roughly half of all observed promoter traps occurred within the 5′-UTR region of transcripts for both the non-IRES and the IRES-containing promoter traps. The IRES-containing promoter trap construct was anticipated to, and may have, functioned as an internal ribosomal entry site for expression of AmCyan, and while the RNASeq data were unable to confirm this, it is possible that the IRES still functioned correctly, however the overall levels of transcription for AmCyan fusion events was too low to detect IRES-containing transcriptional events (only 17 of the 200 positive promoter trap events could be identified using direct RNASeq sequencing). Instead, the RNASeq dataset demonstrated that of the 10 putative 3′-SA sites from the *piggyBac* right end through to the +1 ATG of AmCyan, one 3′-SA site dominated, which was 5′-ATATCGTTAGTCTTTCAACAG-3′. For the promoter trap element not containing the IRES element, the planned splice acceptor 5′-TTCCCCCCTCCCAGCAG-3′ functioned effectively, suggesting that the 5′-ATATCGTTAGTCTTTCAACAG-3′ splice junction -15 bp from the +1 of the AmCyan ORF may be a preferred splice acceptor for a non-sequence related reason, such as the secondary structure of the IRES element. To obtain additional evidence that the AmCyan proximal 3′-SA was not preferred due to a sequence preference, we analyzed the consensus of 42,921 splice acceptors (21 nucleotides upstream of the *ab initio* predicted sites) in the *An. stephensi* SDA500 genome using the standalone version of weblogo (v 2.8.2) (Schneider and Stephens 1990; Crooks *et al.* 2004). The scoring matrix based (Table S4) on base conservation by position yielded a score of 5.18 and 5.23, for the unplanned and the planned 3′-SAs, respectively out of a maximum possible score of 5.67. Thus, while the AmCyan proximal 3′-SA was preferred for splicing, it was not computationally predicted to be more efficient, suggesting that the IRES element may play a role in the preferred splicing of the promoter trap.

A surprising event for the promoter trap elements was the preference for similar gene insertion. The 15 nt upstream tag on the IRES-containing promoter trap element allowed for us to distinguish between the two elements and confirm at both the DNA level (splinkerette) and the RNA level (RNASeq) that gene trap elements, initiating from different start locations within the genome, independently landed into nearby TTAA sites within the same intron within the same gene.

### Tissue-type preference

Reported promoter traps were predominantly found in metabolically-active tissues, such as the fat body, muscles, and imaginal discs. The promoter trap construct will report only when expressed, this metabolically-active tissues may allow for greater expression of the AmCyan reporter. As such, the promoter trap may be easier to report when inserted into genes with greater levels of gene expression in highly metabolic tissues. The identification of putative promoters within the fat body, imaginal discs, and neural tissues propose interesting tools for future studies for the functional analysis of genes. For example, the fat body is a highly metabolic tissue that could be used to express and export proteins of interest into the hemolymph of *An. stephensi*, as previously identified in *Aedes aegypti* for a carboxypeptidase gene (Moreira *et al.*, 2000) and a vitellogenin gene (Kokoza *et al.*, 2000). Promoters for genes active in the imaginal discs could be used to drive genes to interrogate questions regarding development, or to conduct misexpression studies. Promoters identified to have neural expression patterns could be used to drive genes expressing modified genes encoding targets for insecticides to investigate the functional analysis of SNPs within insecticide resistant alleles.

### IRES *vs.* non-IRES

In our study, we found that the IRES and non-IRES containing promoter traps behaved similar to each other with respect to insertion site preference within transcript. Given that previous studies have identified that *piggyBac* transposon has a preference to insert into the 5′-end of transcripts (Häcker *et al.* 2003; Bonin and Mann 2004; Wilson *et al.* 2007; Wang *et al.* 2008; Liang *et al.* 2009), it is possible that the behavior of the IRES and non-IRES containing promoter trap elements is due to the nature of *piggyBac*. In addition, however, we were unable to determine at the RNA-level if the IRES element was functional in our promoter trap due to an unanticipated 3′-splice acceptor -15 bp upstream of the AmCyan open reading frame, thus it is likely that the IRES element was spliced out of the transcript and could not be tested for functionality. Future approaches could include modification of the terminal AG of the splice acceptor sequence for the additional putative splice acceptor elements identified upstream of the AmCyan open reading frame in the IRES-containing construct. Differences regarding local hopping and random remobilization between the IRES and non-IRES containing promoter traps was observed, however it is known that the insertion start location highly influences the remobilization of the transposable element (Esnault *et al.*, 2011).
